# Whole Genome Sequencing of *Kodamaea ohmeri* SSK and Its Characterization for Degradation of Inhibitors from Lignocellulosic Biomass

**DOI:** 10.3390/biology14050458

**Published:** 2025-04-24

**Authors:** Yong-Qiang Yang, Xu Li, Zhi-Fei Wang, Yu-Long Deng, Zhen-Zhi Wang, Xing-Yu Fang, Mao-Dong Zhang, Wei Sun, Xin-Qing Zhao, Zhi-Qiang Liu, Feng-Li Zhang

**Affiliations:** 1 School of Life and Health Sciences, Hainan University, Haikou 570228, China; 22210710000017@hainanu.edu.cn; 2 State Key Laboratory of Microbial Metabolism, Joint International Research Laboratory of Metabolic & Developmental Sciences, School of Life Sciences and Biotechnology, Shanghai Jiao Tong University, Shanghai 200240, China; 13280757366@163.com (X.L.); hata.you@sjtu.edu.cn (Z.-F.W.); 18294595743dyl@sjtu.edu.cn (Y.-L.D.); 2022350109wzz@sjtu.edu.cn (Z.-Z.W.); zmd0426@sjtu.edu.cn (M.-D.Z.); bacterior@sjtu.edu.cn (W.S.); xqzhao@sjtu.edu.cn (X.-Q.Z.); 3 School of Electronic Information and Electrical Engineering, Shanghai Jiao Tong University, Shanghai 200240, China; yuri_cang@sjtu.edu.cn

**Keywords:** detoxification enzyme, inhibitor detoxification, lignocellulosic biomass, *Kodamaea ohmeri*, strain tolerance, whole genome sequencing

## Abstract

Lignocellulosic biomass is widely recognized as a renewable resource for bioconversion. However, the presence of inhibitors such as furfural, 5-HMF, and acetic acid can inhibit cell growth, thereby affecting the overall efficiency of the bioconversion process. The studies on the degradation of lignocellulosic hydrolysate inhibitors by *Saccharomyces cerevisiae* have been limited. In this research, a yeast strain *Kodamaea ohmeri* can degrade inhibitors furfural, 5-HMF, and acetic acid, and the genome sequence of the strain was analyzed. Furthermore, the molecular detoxification mechanism of *K. ohmeri* SSK against lignocellulosic hydrolysate inhibitors was predicted using whole genome sequencing. Annotation based on the COG/KEGG databases identified 57 key detoxification genes, including the alcohol dehydrogenase (ADH) gene, aldo-keto/aldehyde reductase (AKR/ARI) gene, and aldehyde dehydrogenase (ALDH) gene. Stress tolerance experiments revealed that the maximum tolerance concentration for the strain was 5.2 g/L of furfural, 2.5 g/L of 5-HMF, and 5.9 g/L of acetic acid, respectively. A NAD(P)^+^-dependent bifunctional enzyme with possible ADH and ARI activities was found by conserved domain analysis. Phylogenetic analysis indicated that this enzyme shared 99% homology with the detoxification enzyme from *S. cerevisiae* S288C (GenBank: Q04894.1). This study represents the first comprehensive analysis of the inhibitor detoxification network in *K. ohmeri* SSK from a genome perspective, providing theoretical targets and design strategies for developing highly efficient biorefinery strains.

## Simple Summary

In this study, we predicted the molecular detoxification mechanisms of *Kodamaea ohmeri* in response to inhibitors including furfural, 5-hydroxymethylfurfural (5-HMF), and acetic acid. This investigation was conducted through whole genome sequence analysis and fifty-seven key detoxification genes (e.g., ADH, AKR, and ALDH) encoded proteins possibly involved in inhibitor degradation were analyzed. The maximum tolerance concentration of the strain to furfural, 5-HMF, and acetic acid was 5.2, 2.5, and 5.9 g/L, respectively. These results provide valuable cell candidates for the utilization of lignocellulosic biomass and efficient biorefinery.

## 1. Introduction

*Kodamaea ohmeri* is a species of yeast within the family *Saccharomycetaceae*, and belongs to the genus *Kodamaea*, which was previously classified as *Pichia ohmeri* or *Yamadazyma ohmeri* [1]. The genus *Kodamaea* includes five known species: *K. anthrophila*, *K. kakaduensis*, *K. laetipori*, *K. nitidulidarum*, and *K. ohmeri*. *K. ohmeri* is widely distributed, and its strain characteristics are influenced by the source of isolation. Strains isolated from clinical samples are considered conditionally pathogenic, while those from natural environments or food sources are commonly used in the food industry for fermentation purposes [2,3]. *K. ohmeri* BG3 isolated from the intestine of the marine fish *Hexagrammes otakii* can produce phytase [4]; *K. ohmeri* NH-9, isolated from honeycomb and flower samples, can produce D-arabinitol [5]; and *K. ohmeri* W5 isolated from fermented soybean paste can produce phenyllactic acid [6]. *K. ohmeri* strains have also been isolated during the fermentation of cocoa beans [7], in the production of Brazilian cheese and fermented sufu paste [8,9], and in the brewing of Hakka rice wine and Cabernet Sauvignon wine [10,11].

Lignocellulosic biomass derived from grasses, wood, crop residues, and municipal waste serves as a rich source of renewable sugars [12]. During the sugar extraction process, lignocellulose undergoes dilute acid treatment at high temperatures and high pressures, resulting in the formation of inhibitory by-products such as furfural and acetic acid [13,14]. The presence of these inhibitors negatively impacts the structural integrity and functionality of microbial cell membranes, thereby reducing their viability in lignocellulosic hydrolysate [15]. Researchers have elucidated the tolerance mechanisms of *K. ohmeri* to these inhibitors; specifically, and the *K. ohmeri* can tolerate 3 g/L of 5-HMF, 0.65 g/L of furfural, and 6 g/L of acetic acid, respectively [16]. This tolerance is attributed to its capacity for efficient degradation of furanal—an important component among lignocellulose-derived microbial inhibitory compounds (LDMICs) that can severely hinder microbial growth, and negatively impact microbial fermentation processes. The effective detoxification of furanal and acetic acid by *K. ohmeri* significantly mitigates the inhibitory effects on its own growth and metabolism, while paving new pathways for the efficient utilization of lignocellulosic hydrolysates in biochemical production [17].

Several studies have reported the identification of related degradation pathways and genes in microorganisms. In 1969, Trudgill described the furfural degradation pathway in *Pseudomonas putida* F2, which was later confirmed by Koenig, Koopman, and others [18,19,20]. Furthermore, Koopman identified a novel HMF/furfural oxidoreductase from *Cupriavidus basilensis* HMF14, which catalyzes the conversion of HMF to 2,5-furanedicarboxylic acid [21], thereby establishing a connection between the HMF degradation pathway and the furfural degradation pathway. The furfural degradation pathway, illustrated in Figure S1, initiates with the reduction of furfural to furanol. This is followed by oxidation to furanal and further oxidation to furanic acid. The degradation products of HMF, along with furanic acid generated from furfural oxidation, enter the subsequent degradation pathway and ultimately flow into the tricarboxylic acid (TCA) cycle. The bioconversion process that transforms furfural into furanol, subsequently into furanal, and ultimately into furanic acid, represents the crucial microbial activity involved in the detoxification of inhibitors [22].

*K. ohmeri* has attracted attention in genomic studies; however, its potential for lignocellulosic inhibitor detoxification remains largely unexplored. Previous whole-genome sequencing efforts, including the clinical isolate KO20 [23] and strains derived from honeybee midguts [24], have primarily focused on niche-specific traits such as gut metabolism or pathogenicity. In contrast, applications in food fermentation have highlighted its ability to produce phytase, D-arabinitol, and phenyllactic acid [4,5,6]. Nevertheless, critical gaps persist in the molecular mechanisms involved in degrading lignocellulosic inhibitors like furfural, 5-HMF, and acetic acid. To date, no studies have annotated genes associated with these pathways or linked genomic features to proteomic functions under inhibitor stress in *K. ohmeri*. Furthermore, there has been a lack of comparative analyses of its detoxification machinery to model organisms like *Saccharomyces cerevisiae*. To address these gaps, this study integrates whole-genome sequencing with COG/KEGG functional annotation, and stress tolerance assays to systematically predict 57 detoxification genes (e.g., ADH, AKR/ARI, and ALDH) and elucidate their possible roles in inhibitor detoxification. This research provides a molecular foundation for understanding the survival strategies employed by *K. ohmeri* SSK in inhibitory environments, and offers valuable insights for its application within the biorefinery industry.

## 2. Materials and Methods

### 2.1. Experimental Strain

The *K. ohmeri* SSK strain (CCTCC No: M20241008) used in this study was provided by the Industrial Microbiology and Bioprocess Engineering Laboratory (IMBE), Shanghai Jiao Tong University. The frozen strain was initially inoculated onto YPD agar medium (the recipe is detailed in Section 2.2), and incubated at 30 °C for 24 h. Single colonies were subsequently isolated and transferred to a YPD liquid medium, where they were incubated at 30 °C with shaking at 200 rpm for 18 h. This culture served as the seed solution for subsequent experiments.

### 2.2. Culture Medium

YPD medium composition (g/L): yeast extract 10, peptone 20, glucose 20, pH adjusted to 7 with NaOH, and 20 g/L of agar added for solid medium. All the reagents for this method were purchased from Sangon Biotech Co., Ltd., Shanghai, China.

To investigate the effects of various inhibitor concentrations on the growth of the strains, two types of media were designed: single inhibitor medium and mixed inhibitor medium. Single inhibitor medium: furfural (Meryer Chemical Technology Co., Ltd., Shanghai, China) (0.74, 1.82, 3.21, 6.39 g/L), 5-HMF (Meryer Chemical Technology Co., Ltd., Shanghai, China) (0.37, 0.69, 1.34, 2.48 g/L), and acetic acid (Sangon Biotech Co., Ltd., Shanghai, China) (2.33, 5.93, 10.88, 21.31 g/L) were added to the basal YPD medium at different concentration gradients corresponding to the levels normally present in lignocellulosic hydrolysates. Mixed inhibitor medium: three inhibitors with different concentration gradients (e.g., furfural 0.74 g/L + 5-HMF 0.37 g/L + acetic acid 2.33 g/L) were simultaneously added to the basal YPD medium. These concentration gradients were categorized as low (C_low_), medium–low (C_mid-low_), medium–high (C_mid-high_), and high (C_high_) based on increasing concentrations from low to high.

### 2.3. Kinetics of Strain Growth and Inhibitor Detoxification

The logarithmic phase seed culture was inoculated into inhibitor-containing YPD medium at a 5% (*v*/*v*) concentration and incubated at 30 °C with shaking at 200 rpm for 72 h. Samples were collected every 12 h. Three biological replicates were performed, with the inhibitor-free YPD medium serving as a blank control.

Biomass determination: A—0.2 mL aliquot of the culture was transferred to each cell of a 96-well plate, and the optical density (OD) at 600 nm was measured using a Multiskan GO (Thermo Fisher Scientific, Waltham, MA, USA).

Inhibitor concentration analysis: The remaining culture was centrifuged at 8000 rpm for 3 min at 4 °C. The supernatant was filtered through a 0.22 μm membrane and analyzed for residual inhibitor concentrations by HPLC instrument (Waters Alliance e2695 HPLC, Waters, Milford, MA, USA) with a Bio-Rad Aminex^®^ HPX-87H column (Bio-Rad, Hercules, CA, USA). The elution condition was controlled at 50 °C, 4 mM sulfuric acid, and 0.6 mL/min flow rate [25].

### 2.4. Genome DNA Extraction and Sequencing

Genomic DNA was extracted using the EZ-10 Centrifuge Column Plant Genomic DNA Purification Kit (No. B518261, Shanghai Biotechnology, Shanghai, China). The integrity of the DNA was confirmed by agarose gel electrophoresis, and the concentration and quality were assessed using a Nanodrop 2000/Qubit.

DNA was fragmented to 100–500 bp using NEB’s DNA Fragmentase, followed by end-repair to generate blunt ends with phosphate groups. The 3′ ends were then A-tailed. The library fragment size was optimized using T4 DNA ligase, followed by PCR amplification with high-fidelity polymerase and the Agencourt SPRIselect Kit (Beckman Coulter, Miami, FL, USA). The quality of the library was assessed using Qubit (Thermo Fisher Scientific, Waltham, MA, USA) and the Agilent 2100 Bioanalyzer (Agilent Technologies, Santa Clara, CA, USA), ensuring a DNA concentration of more than 5 ng/μL and a fragment length primarily ranging from 300 to 400 bp. Finally, the library was subjected to high-throughput sequencing using 2 × 150 bp paired-end sequencing on the Illumina platform, generating high-quality FastQ data.

### 2.5. Sequencing Data Accusation and Assembly

Sequencing data quality control was performed using FastQC (v 0.11.5), and sequencing data were filtered using TrimGalore (v 0.4.4) to remove splice sequences from reads, trim the ends of reads with a quality value of less than 20, and discard reads containing invalid or ambiguous bases and greater than 50 bp in length. Sequencing errors were corrected by error-correcting quality-corrected reads using the BBTools Program [26]. Overlapping clusters were assembled using SPAdes (v 3.11.1) [27], and Kraken was employed for sorting and filtering. Gaps in the assembly were filled, base corrections were made, and overlapping clusters were combined into scaffolds using GapCloser (v 1.12) [28]. Finally, QUAST (v 4.6.1) was utilized for comprehensive quality assessment to ensure the high quality, accuracy, and completeness of the genome sequence [29].

### 2.6. Gene Prediction and Annotation

The RNAmmer (v 1.2) and tRNAscan-SE (v 2.0) software were used to predict rRNAs and tRNAs in the genome, respectively [30,31]. GeneMark-ES (v 4) was employed for the prediction of eukaryotic genes [32]. The predicted nucleotide sequence of the gene was then translated into an amino acid sequence using Transeq (v 6.6.0.0) software and compared using BLASTP (v 0.9.18) against the COG, NR (the non-redundant protein database), STRING, and Swiss-Prot databases to obtain preliminary functional information. Gene Ontology (GO) annotation was performed using Blast2GO (v 2.5.0) to classify the genes functionally. Additionally, the BLASTP algorithm was used to compare the sequences with the KEGG (Kyoto Encyclopedia of Genes and Genomes) database to obtain KO numbers, allowing the determination of the biological pathways involved in the genes. Finally, a phylogenetic tree was constructed using Mega7.0 software to analyze the functions of the screened genes [33].

### 2.7. Statistical Analyses

The experimental results of cell growth and inhibitor detection were analyzed by GraphPad Prism 9.5 software and expressed as the mean values ± standard deviation (SD). Differences between the mean values were considered significant at a *p*-value of < 0.001. Origin 2021 software was used for drawing figures.

## 3. Results

### 3.1. Growth Kinetics and Detoxification Characteristics of K. ohmeri SSK Under Stress of Inhibitors

**Figure 1:**
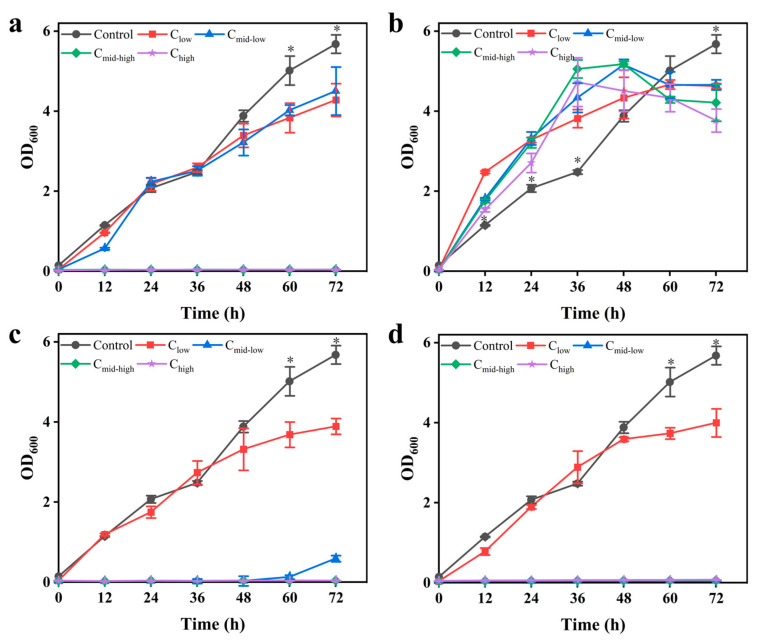
The growth curves of *K. ohmeri* SSK in YPD at varying concentrations of furfural (**a**), 5-HMF (**b**), AA (**c**), and mixed inhibition (**d**) are presented. The data presented here are the mean values ± SD calculated from three independent replicates. (* *p* < 0.001 between control and C_low_).

The stress response of *K. ohmeri* SSK strain was evaluated by gradually adding furfural, 5-HMF, acetic acid, and their combined inhibitors to the YPD medium (Figure 1). The experimental results revealed that the maximum biomass (OD_600_ = 4.5 ± 0.6) was highly significantly different from that of the control group when the value of furfural concentration was 0.74 g/L or 1.82 g/L (medium–low-concentration group). The cell growth was completely inhibited when the value of furfural concentrations was 3.21 g/L (medium–high- and high-concentration groups) (Figure 1a). The biomass of three concentration groups (0.37–2.48 g/L of 5-HMF) reached 82–91% of the control group, suggesting that the strain possibly acclimated to stress through metabolic adaptation (Figure 1b). The maximum biomass of the low-concentration group with 2.33 g/L of acetic acid declined to 68.5 ± 3.7% of that in the control group, while the medium–low-concentration group with 5.93 g/L of acetic acid showed growth retardation of 48 h. Cell growth was entirely inhibited when the value of acetic acid concentrations was 10.88 g/L (Figure 1c). The biomass accumulation in the low-concentration mixed inhibitor group (0.74 g/L of furfural, 0.37 g/L of 5-HMF, and 2.33 g/L of acetic acid) was reduced by 42%. However, the OD_600_ reached 3.99 ± 0.35 at 72 h. In contrast, the medium–low-concentration mixed inhibitor group exhibited growth stagnation, indicating that the synergistic effect of multi-factorial stress enhanced toxicity (Figure 1d).

**Figure 2:**
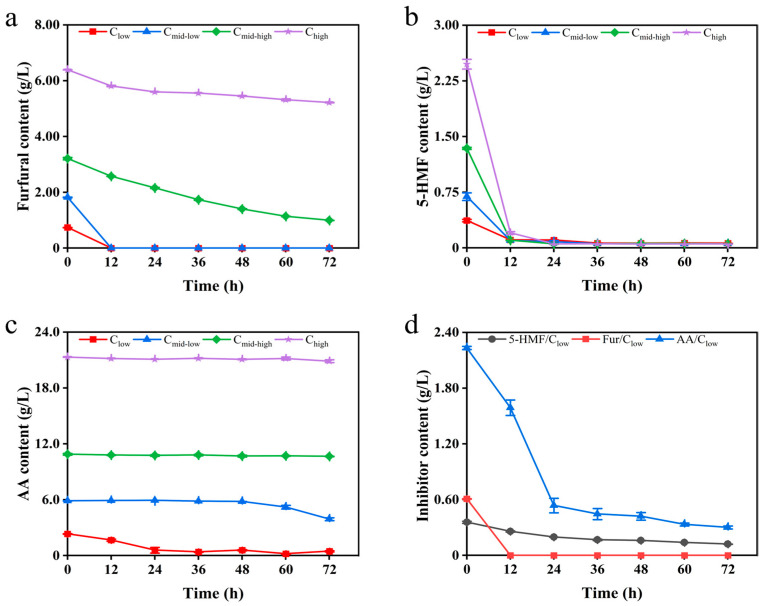
Degradation of different concentrations of furfural (**a**), 5-HMF (**b**), AA (**c**), and mixed inhibition (**d**) by *K. ohmeri* SSK in YPD. The data presented here are the mean values ± SD calculated from three independent replicates.

The detoxification capabilities of inhibitors by *K. ohmeri* SSK were evaluated. Under furfural stress, detoxification was nearly complete within 12 h at low and medium–low concentrations. In contrast, the growth of *K. ohmeri* SSK was completely inhibited at medium–high and high concentrations. Notably, despite the complete inhibition of biomass growth, furfural continued to be degraded; its concentration significantly decreased to low levels after 72 h of fermentation under medium–high-concentration conditions (Figure 2a, Figure S3a). Different concentrations of 5-HMF were almost completely degraded by the strain within 12 h of the reaction (Figure 2b, Figure S3b). The cell growth was delayed for an additional 48 h before initiating the degradation process at medium–low-concentration sample; and cell growth was completely inhibited when cells were at medium–high concentrations and high concentrations of acetic acid (Figure 2c, Figure S3c). In the mixed inhibitor system, the degradation pattern of each inhibitor component was similar to that observed in the single inhibitor system at low concentrations. The mixed inhibitors did not enhance the inhibitory effect. However, when the mixed inhibitor concentration exceeded the low concentration condition, the growth of the strain was completely inhibited (Figure 2d, Figure S3d).

### 3.2. Whole Genome Sequencing of K. ohmeri SSK

The genome sequence data of *Kodamaea ohmeri* SSK have been deposited in the NCBI GenBank database under Bioproject PRJNA1241482, Biosample SAMN47558000, and accession number JBMOUP000000000. The statistics of the final assembly results are shown in Table 1.

**Figure 3:**
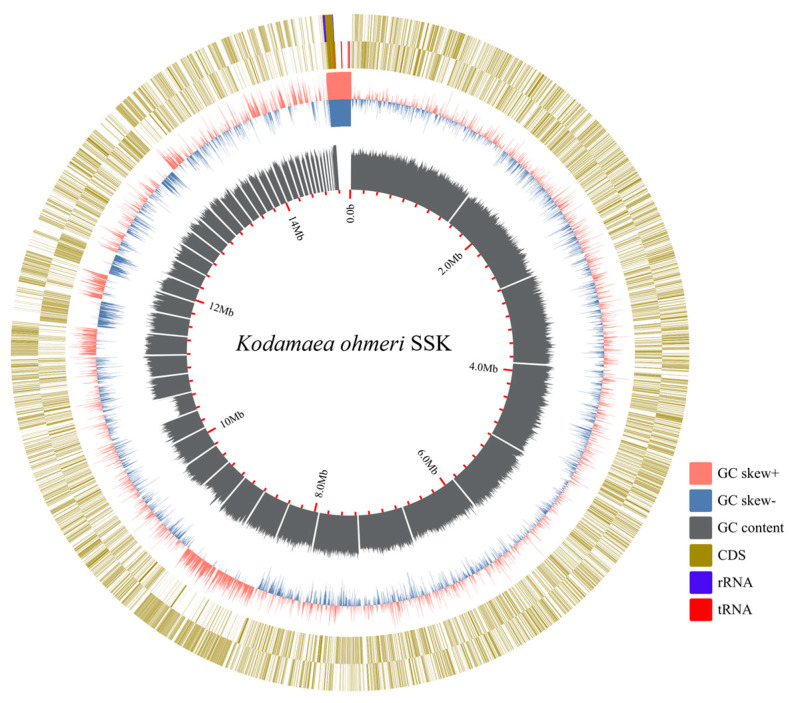
Genome circle diagram of strain *K. ohmeri* SSK. The innermost circle denotes the scale of genome size from the interior to the exterior. The second circle indicates the GC content, while the third circle denotes the GC skew, with a relative predominance of G content over C content towards the exterior, and vice versa towards the interior. The fourth and fifth circles represent the coding sequences, wherein tRNAs and rRNAs are distributed.

A genomic circular map was constructed based on the collected data (Figure 3). The total number of predicted genes in the genome of *K. ohmeri* SSK is 6328, which includes three hundred thirty-nine transfer RNAs (tRNAs) and seven ribosomal RNAs (rRNAs) (comprising 5S rRNAs, and one each of 16S and 23S rRNAs). The average gene length is 1665.41 bp, resulting in a gene density of 0.42 genes per thousand base pairs, indicating a relatively sparse distribution of genes throughout the genome. The GC content within the gene regions accounts for 70.44% of the total genome, while the non-gene (intergenic) regions constitute 29.56%. The GC content of intergenic regions is 36.96%, which is notably lower than that of the gene regions.

Comparative genomics revealed that *K. ohmeri* SSK possesses the largest genome (14.3 Mb) and the highest GC content (43.34%) among sequenced strains, with amplification of detoxification genes (e.g., ADH, ALDH, and ARI) likely driven by its ecological niche in the bamboo forest. In contrast, clinical isolates (e.g., KO20) have smaller genomes with no detoxification genes annotated, reflecting distinct evolutionary pressures (Table 2). These genomic differences highlight the suitability of *K. ohmeri* SSK for lignocellulosic biorefinery applications. Such features may enhance its ability to degrade furfural, 5-HMF, and acetic acid, as validated by our stress tolerance assays.

### 3.3. Gene Functional Annotation

**Figure 4:**
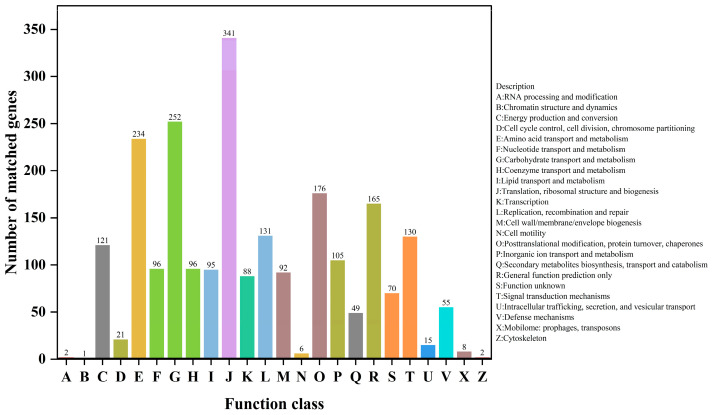
COG functional classification map of *K. ohmeri* SSK.

The functional annotation of the coding genes in *K. ohmeri* SSK was performed using the COG and KEGG databases to elucidate their biological roles. Among 6328 coding genes, 2595 were successfully annotated, with 2351 genes classified into 24 functional categories (Figure 4). Notably, categories W (extracellular structures) and Y (nuclear structure) were absent, indicating that these functions are non-essential for *K. ohmeri* SSK. The most abundant categories include translation, ribosomal structure, and biogenesis (341 genes), followed by carbohydrate transport and metabolism (252 genes), and amino acid transport and metabolism (234 genes). This analysis result suggests that the strain exhibits robust metabolic activity related to carbohydrate and amino acid metabolism, indicating the presence of a comprehensive metabolic pathway that efficiently facilitates the uptake, transport, and metabolism of these essential nutrients. Consequently, this provides a foundation for cell growth, energy supply, and material synthesis. Additionally, category C (energy production and conversion) has 121 annotations; this indicates that *K. ohmeri* SSK possesses genes involved in intracellular energy production and conversion. In conclusion, such findings suggest that the strain can maintain intracellular energy balance while supporting a wide range of energy-consuming physiological processes essential for cellular activities.

**Figure 5:**
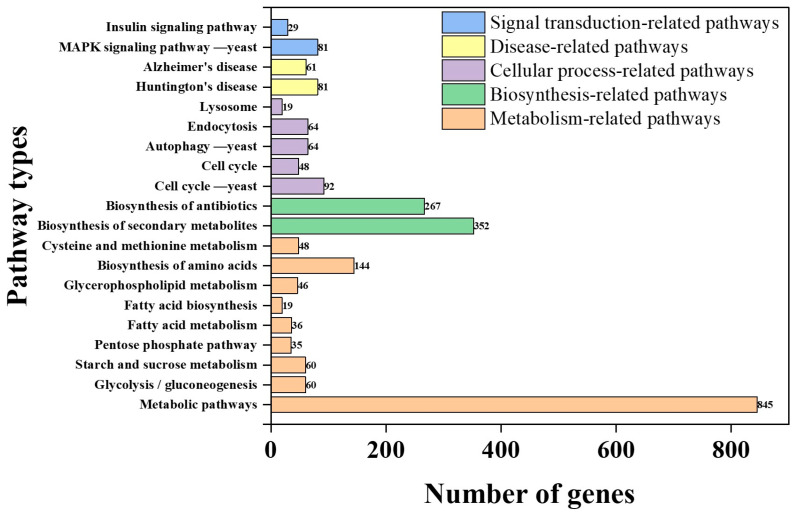
The association map between biological pathway categories and gene quantities.

Out of the 8070 protein-coding genes, 845 genes are associated with metabolic pathways, highlighting the pivotal role of metabolism in cellular functions (Figure 5). Key pathways include secondary metabolite biosynthesis (352 genes) and antibiotic biosynthesis (267 genes), suggesting the strain’s potential to produce functional secondary metabolites and antibiotics, which may enhance environmental adaptability and microbial competition. However, annotations for polyketide glycoconjugate biosynthesis and vancomycin antibiotic biosynthesis are limited.

The annotation results highlight the robust metabolic capabilities of *K. ohmeri* SSK, particularly in carbohydrate and amino acid metabolism, as well as its potential for secondary metabolite production.

### 3.4. Screening of Furfural and 5-HMF Degradation-Related Genes

Furfural and 5-HMF, two critical inhibitors in lignocellulosic hydrolysates, share overlapping detoxification pathways [34]. Both compounds are oxidized to furoic acid via aldehyde dehydrogenase (ALDH)-mediated catalysis. ALDH binds to aldehyde substrates via cysteine residues in the active site (e.g., Cys249), forming covalent intermediates that ultimately yield carboxylic acid products. This catalytic process involves the “aromatic box” structure within the substrate-binding domains (e.g., residues Lys192, Glu268, etc.) [34]. While 5-HMF undergoes additional oxidoreductase-driven conversion to 2,5-furandicarboxylic acid before decarboxylation to furoic acid [35]. Ultimately, furoic acid is assimilated into the TCA cycle as 2-ketoglutarate, thereby linking inhibitor detoxification to central energy metabolism [36].

Genomic analysis of *K. ohmeri* SSK identified 53 genes encoding enzymes critical for furfural/5-HMF detoxification, which can be categorized into three functional classes (Table 3). Alcohol dehydrogenases (ADH, thirty-one genes): This group includes eight zinc-dependent, sixteen NAD(P)^+^-dependent short-chain ADHs, and seven variants (e.g., arabinose ADH). These enzymes catalyze the NAD(P)^+^-driven reduction of furfural to less toxic furanol; Aldo-keto/aldehyde reductase (AKR/ARI, fourteen genes): This category comprises twelve AKR and two ARI that reduce aldehyde groups (-CHO) to hydroxyls (-OH) via NAD(P)^+^-dependent mechanisms; Aldehyde dehydrogenases (ALDH, eight genes): This group features six NAD⁺-dependent ALDH and two semialdehyde dehydrogenases that oxidize furfural/5-HMF into furoic acid.

The genomic repertoire of *K. ohmeri* SSK highlights its evolutionary adaptation to inhibitor-rich environments. Functional redundancy across ADH, AKR/ARI, and ALDH families ensures a robust detoxification capacity that facilitates efficient conversion of furfural/5-HMF into metabolically compatible intermediates. This genetic architecture underscores the strain’s potential for applications in lignocellulosic bioconversion.

### 3.5. ADH Sequence Analysis

The amino acid sequence of the known alcohol dehydrogenase (ADH) from *S. cerevisiae* S288C was subjected to homology analysis with the ADH encoded in the genome of *K. ohmeri* SSK (scaffold17;4744_g and scaffold1;197_g), as well as with those from molds and bacteria. Phylogenetic trees were constructed using Mega7.0 software and Bootstrap analysis (1000 replicates), ensuring branching reliability (Figure S4). The result revealed that the ADH encoded by scaffold17;4744_g exhibits a much closer evolutionary relationship with the ADH encoded by scaffold1;197_g and the ADH of *S. cerevisiae* S288C. Notably, these related sequences show a high degree of similarity, reaching 99%. Furthermore, substantial similarity was observed between these ADHs and those from two bacterial species. The conserved nature of ADH is reflected in the high retention of its core functional domains [37]. It has been demonstrated that plant and fungal ADH usually contains three key structural domains, namely the ADH_N structural domain with GroES folding, Rossmann folding (NAD(P)^+^ binding site), and zinc-binding site. These structures are highly conserved in yeast, plants, and bacteria [38]. These findings further validate the ADH in *K. ohmeri* SSK, highlighting its possible functional and evolutionary conservation across different organisms.

Building on the homologous evolutionary tree, a comparative analysis of the amino acid sequence of ADH was conducted, followed by a prediction of the protein’s secondary structure for scaffold17;4744_g using SWISS-MODEL (https://swissmodel.expasy.org, accessed on 21 January 2025). The results revealed that the protein encoded by scaffold17;4744_g displays a diverse array of secondary structure types, including eleven α-helices, eighteen β-strands, and five random coils (η), which are alternately distributed throughout the sequence (Figure S5). Notably, within the region encompassing amino acids 210–270, secondary structures such as β11, α6, β12, α7, α8, and β13 appear sequentially. This structural arrangement is likely to enhance the protein’s capability to adapt to varying cellular environments and physiological demands.

### 3.6. Mining of Functional Genes of Acetic Acid Degradation Pathway

In yeasts, the metabolic pathway for acetic acid is primarily catalyzed by acetyl coenzyme A synthase (ACS), which facilitates the conversion of acetic acid and coenzyme A into acetyl coenzyme A. This intermediate then enters the TCA cycle, accompanied by the hydrolysis of ATP [39,40]. According to the COG classification system, this process falls under carbohydrate transport and metabolism (Table 4). Notably, the gene encoding the ACS enzyme is designated as the ACS gene. In *K. ohmeri* SSK, four protein-coding sequences were screened through COG functional annotation: scaffold3;1501_g, scaffold6;2737_g, scaffold6;2738_g, and scaffold26;5535_g, all of which belong to the same ACS superfamily. When ACS was overexpressed, it was found to enhance the binding of acetic acid to coenzyme A, thereby reducing the concentration of acetic acid and improving the strain’s growth in an acetic acid-rich environment [41].

## 4. Discussion

Lignocellulosic biomass, such as corn stover, is rich in fermentable sugars, but the inhibitors (aldehydes and organic acids) in corn stover hydrolysate (CSH) can adversely affect fermentation strains. In this study, a set of concentration gradients of inhibitors was determined based on the prepared actual CSH. The concentrations of inhibitors detected in the actual CSH were 9.20 g/L acetic acid, 2.44 g/L furfural, and 1.28 g/L 5-HMF. The glucose concentration of the 4-fold dilution of the actual CSH was approximately 20 g/L, which meets the carbon requirements of the strain while simultaneously reducing inhibitor concentrations to levels within its tolerance threshold. Specifically, these thresholds were determined to be 2.33 g/L for acetic acid, 0.74 g/L for furfural, and 0.37 g/L for 5-HMF.

In summary, *K. ohmeri* SSK showed remarkable tolerance to various inhibitors, with maximum tolerated concentrations of furfural (5.2 g/L), 5-HMF (2.5 g/L), and acetic acid (5.9 g/L) (HPLC spectra for these species are shown in Figure S2). The furfural tolerance level observed in *K. ohmeri* SSK was higher than those in *Enterobacter hormaechei* UW0SKVC1 (3.3 g/L), *S. cerevisiae* NCYC 3451 (3 g/L), *S. cerevisiae* FY10 (2.4 g/L), and *Enterobacter cloacae* GGT036 (3.4 g/L) [42,43,44,45]. Regarding 5-HMF degradation, *K. ohmeri* SSK achieved near-total degradation within 12 h at comparable concentrations; in contrast, *Rhodosporidium toruloides*, *Cryptococcus curvatus*, and *Lipomyces starkeyi* required longer durations of 20 h, 100 h, and 100 h, respectively, to degrade the same compound [46]. This underscores a significant advantage of *K. ohmeri* SSK in terms of its efficiency in degrading 5-HMF. In general, acetic acid concentrations of 1–2 g/L had negligible effects on growth, moderate inhibition was observed at 3–5 g/L, and tolerance was higher at 5–6 g/L. Some strains of *S. cerevisiae* have demonstrated strong tolerance of up to 8–10 g/L of acetic acid, maintaining sustained growth at these concentrations [47,48,49]. In contrast, *K. ohmeri* SSK displayed moderate tolerance towards acetic acid.

A total of 2595 out of 6328 coding genes were annotated, suggesting that *K. ohmeri* SSK may possess a substantial number of genes with unidentified functions that warrant further investigation. It is probable that the genome contains biological functions and mechanisms that remain unexplored. The functional analysis of the genome concerning protein-coding systems and metabolic features revealed that the highest number of annotations were found in category J (translation, ribosomal structure, and biogenesis), comprising 341 annotations [50,51]. This highlights the critical role of protein synthesis-related functions in *K. ohmeri* SSK, necessitating the involvement of numerous genes to ensure proper intracellular protein synthesis while supporting cell growth, metabolism, and various physiological activities.

The rich metabolic pathways of the strain facilitate its efficient utilization of various nutrients, including multiple carbon and nitrogen sources, while also allowing for a flexible response to fluctuations in nutrient composition within the environment. This characteristic aligns with the results of genome analysis of *Pyrodictium delaneyi* Hulk, whose genome encodes a variety of glycosidases and nitrogen assimilation-related genes, which endows it with a unique ability to utilize carbon and nitrogen sources (e.g., nitrate, ammonia) [52]. In addition, the diverse metabolic pathways enable the strain to effectively respond to multiple environmental stresses (e.g., temperature, pH changes, and the presence of toxic substances), and to maintain normal cellular physiological functions and internal environmental homeostasis by dynamically regulating metabolic flow, thus significantly enhancing its competitiveness and adaptability for survival in complex ecological niches. These properties enable them to show important potential applications in bioremediation, industrial fermentation, and other fields [53].

The prediction of 57 genes related to detoxification in *K. ohmeri* SSK, including a potential NAD(P)^+^-dependent bifunctional ADH/ARI enzyme was conducted. This enzyme is functionally similar to ADH6 in *S. cerevisiae*, which catalyzes alcohol oxidation and aldehyde reduction under redox pressure, underscoring its unique adaptability to lignocellulosic inhibitors [54]. Unlike *S. cerevisiae*, which relies on monofunctional enzymes for furfural reduction, this bifunctional enzyme likely enhances metabolic flexibility under redox stress by concurrently catalyzing alcohol oxidation and aldehyde reduction—a critical advantage for industrial hydrolysate detoxification. Structural analysis revealed conserved domains (e.g., AKR_AKR1-5-like, ALDH-SF superfamily) that enable broad substrate specificity, facilitating the reduction of diverse aldehydes (e.g., furfural, 5-HMF) to fewer toxic alcohols [55]. This versatility is further supported by multiple ALDH domains (e.g., ALDH_F1-2_Ald2-like), which may have evolved to optimize survival in inhibitor-rich environments. However, the moderate acetic acid tolerance (5.9 g/L) of *K. ohmeri* SSK’s compared to engineered *S. cerevisiae* strains (8–10 g/L) exposes unresolved limitations. Although the function of ACS genes was predicted, their inefficiency in ATP-dependent acetate assimilation may hinder the acetic acid degradation process. This suggests a need for cofactor engineering (e.g., enhancing NADPH regeneration) or ACS overexpression to alleviate redox imbalances. Furthermore, 58% of *K. ohmeri* SSK’s coding genes remain unannotated, potentially encoding novel detoxification pathways unexplored. For instance, conserved AKR/ALDH domains indicate potential undiscovered aldehyde-metabolizing enzymes, yet their roles require experimental validation (e.g., CRISPR knockouts, proteomics).

Although this study provides a foundational description of the detoxification network of the *K. ohmeri* SSK and genomic predictions highlight *K. ohmeri* SSK’s enzymatic potential, the absence of in vivo validation merits consideration. First, the functional annotation relies on various databases for blastp comparisons (e.g., COG, KEGG, and NR), which may overlook novel pathways encoded by 58% of the unannotated genes. Second, while the role of ADH, AKR/ARI, and ALDH in inhibitor degradation is genetically predicted, they lack experimental validation (e.g., RT-qPCR, knockout/overexpression). Finally, synthetic inhibitor mediators may not fully replicate lignocellulosic hydrolysates and therefore need to be tested in substrates of actual CSH. Future work should incorporate multi-omics approaches to elucidate unannotated gene functions, as well as experimental validation of transcriptional analyses concerning biodegradation processes of furfural, 5-HMF, and acetic acid by *K. ohmeri* SSK. Additionally, validating strain performance within authentic CSH environments is essential. By bridging these gaps, as a naturally tolerant strain, the *K. ohmeri* SSK will serve as a chassis cell factory for industries on the utilization of lignocellulosic biomass.

## 5. Conclusions

Fifty-seven detoxification genes, including a possible bifunctional ADH/ARI enzyme gene from *K. ohmeri* SSK were predicted, and these genes were possibly related to tolerance to lignocellulosic inhibitors. *K. ohmeri* SSK can grow at 5.2 g/L of furfural, 2.5 g/L of 5-HMF, or 5.9 g/L of acetic acid. However, 58% of unannotated genes possibly participated in inhibitors’ metabolism pathways. In future studies, we will integrate multi-omics approaches to annotate these uncharacterized genes and validate the functions of ADH/ARI through CRISPR-based techniques and transcriptomic analysis of *K. ohmeri* SSK.

## Figures and Tables

**Table 1:** Key metrics for the genome assembly of *K. ohmeri* SSK.

Metric	Value
Sample	*K. ohmeri* SSK
Total assembly length	14,959,343 bp
GC content	43.34%
Number of scaffolds	457
Scaffold N50	0.54 Mb
Scaffold L50	8
N’s per 100 kbp	4.29

“N’s per 100 kbp” indicates the count of ambiguous nucleotide bases (N) in every 100,000 bp of the assembled sequence.

**Table 2:** Comparative genomic features of *K. ohmeri* strains.

Strain	Genome Size (Mb)	GC (%)	N50 (Mb)	L50	Number of Contigs	Source
*K. ohmeri* SSK	14.3	43.34	0.5	8	457	Bamboo
*K. ohmeri* 148	12.6	43.00	1.8	3	20	Honeybee
*K. ohmeri* NRRL Y-1932	12.3	42.50	0.29	11	95	NA
*K. ohmeri* W5	12.5	42.50	0.96	5	26	Doubanjiang
*K. ohmeri* UWOPS05-228.2	12.3	42.50	0.76	6	89	Bertam Palm
*K. ohmeri* 3873	12.3	43	0.36	11	104	Homo sapiens
*K. ohmeri* UWOPS01-666b4	12.3	42.50	1.4	4	62	Distimake tuberosus
*K. ohmeri* KO20	12.4	42.5	0.25	16	104	Clinical Blood
*K. ohmeri* R6205-2	12.1	43.00	0.04	90	524	Feces

NA is representative of “Not Available”.

**Table 3:** Genes involved in furfural and HMF degradation in *K. ohmeri* SSK.

Functional Categories	Annotation Description	Number
Alcohol dehydrogenase (31)	Zn-dependent alcohol dehydrogenase	8
	NAD(P)^+^-dependent dehydrogenase, short-chain alcohol dehydrogenase	16
	Other alcohol dehydrogenase	7
Aldehyde reductase, aldo/keto reductase (14)	Aldo/keto reductase	12
	Aldehyde reductase	2
aldehyde dehydrogenase (8)	NAD^+^-dependent aldehyde dehydrogenase	6
	Semialdehyde dehydrogenase	2

**Table 4:** Annotation of ACS superfamily genes in *K. ohmeri* SSK.

Gene	Amino Acid Length	COG Best Hit Length	Alignment Length	Identity (%)	COG Best Hit Description	COG Class
scaffold3;1501_g	500	428	241	29.0	ACS family major facilitator superfamily protein	G
scaffold6;2737_g	518	428	216	28.7	ACS family major facilitator superfamily protein	G
scaffold6;2738_g	582	491	201	25.9	ACS family major facilitator superfamily protein	G
scaffold26;5535_g	530	455	477	24.3	ACS family major facilitator superfamily protein	G

G is representative of “Carbohydrate transport and metabolism”.

## Data Availability

The original contributions presented in this study are included in the article/Supplementary Material. Further inquiries can be directed to the corresponding authors.

## Supplementary Materials

The following supporting information can be downloaded at https://www.mdpi.com/article/10.3390/biology14050458/s1, Figure S1: Metabolic pathways involved in the degradation of furfural and 5-HMF in various strains [22]. Figure S2: HPLC-based standard curves for furfural, 5-HMF, and acetic acid. Figure S3: HPLC-based quantitative analysis of furfural, 5-HMF, and acetic acid during fermentation under single and mixed inhibitor conditions (low concentration). (a: Furfural degradation at time points from 0 to 72 h; b: 5-HMF degradation at time points from 0 to 72 h; c: Acetic acid degradation at time points from 0 to 72 h; d: Mixed inhibition of furfural, 5-HMF, and acetic acid over 72 h). Figure S4: Phylogenetic tree of *K. ohmeri* SSK and multiple strains constructed based on amino acid sequences of alcohol dehydrogenase (ADH). Figure S5: Multiple sequence alignment of amino acid sequences encoding alcohol dehydrogenase (ADH) from different species.
