# Supplementary material for: Whole Genome Sequencing of Kodamaea ohmeri SSK and Its Characterization for Degradation of Inhibitors from Lignocellulosic Biomass
Source: Biology (Basel). 2025 Apr 24;14(5):458. doi: 10.3390/biology14050458 (PMC12109290; doi:10.3390/biology14050458)
Supplement: Supplementary file 1 [file biology-14-00458-s001.zip › biology-3534015-supplementary.pdf]

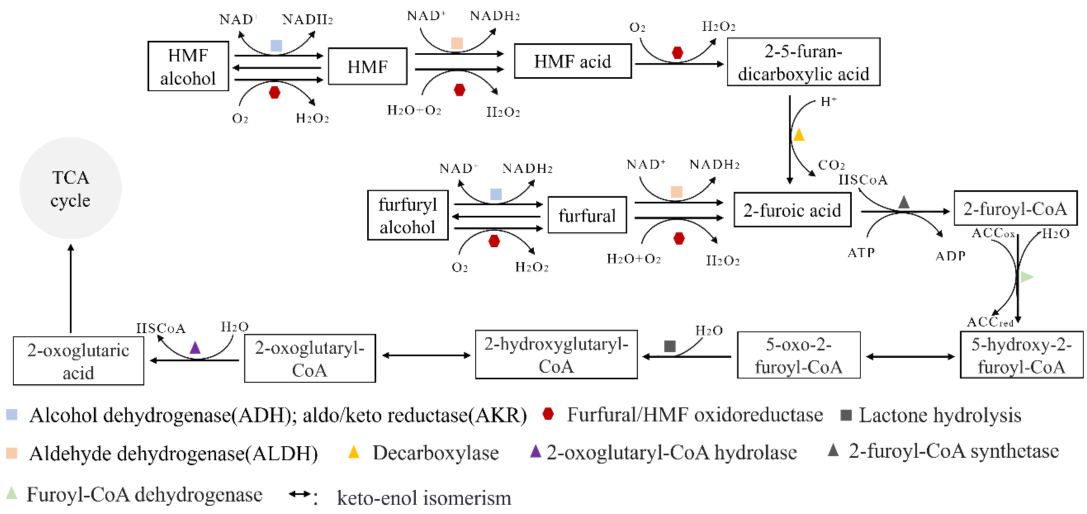

Figure S1: Metabolic pathways of furfural and HMF degradation in strains [22].

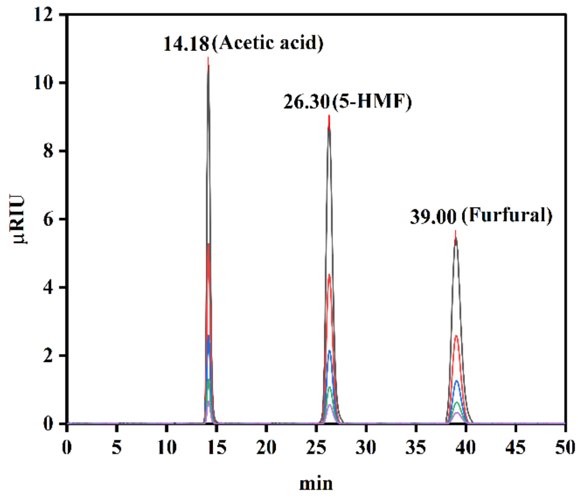

Figure S2: HPLC-based standard curves for furfural, 5-HMF and acetic acid.

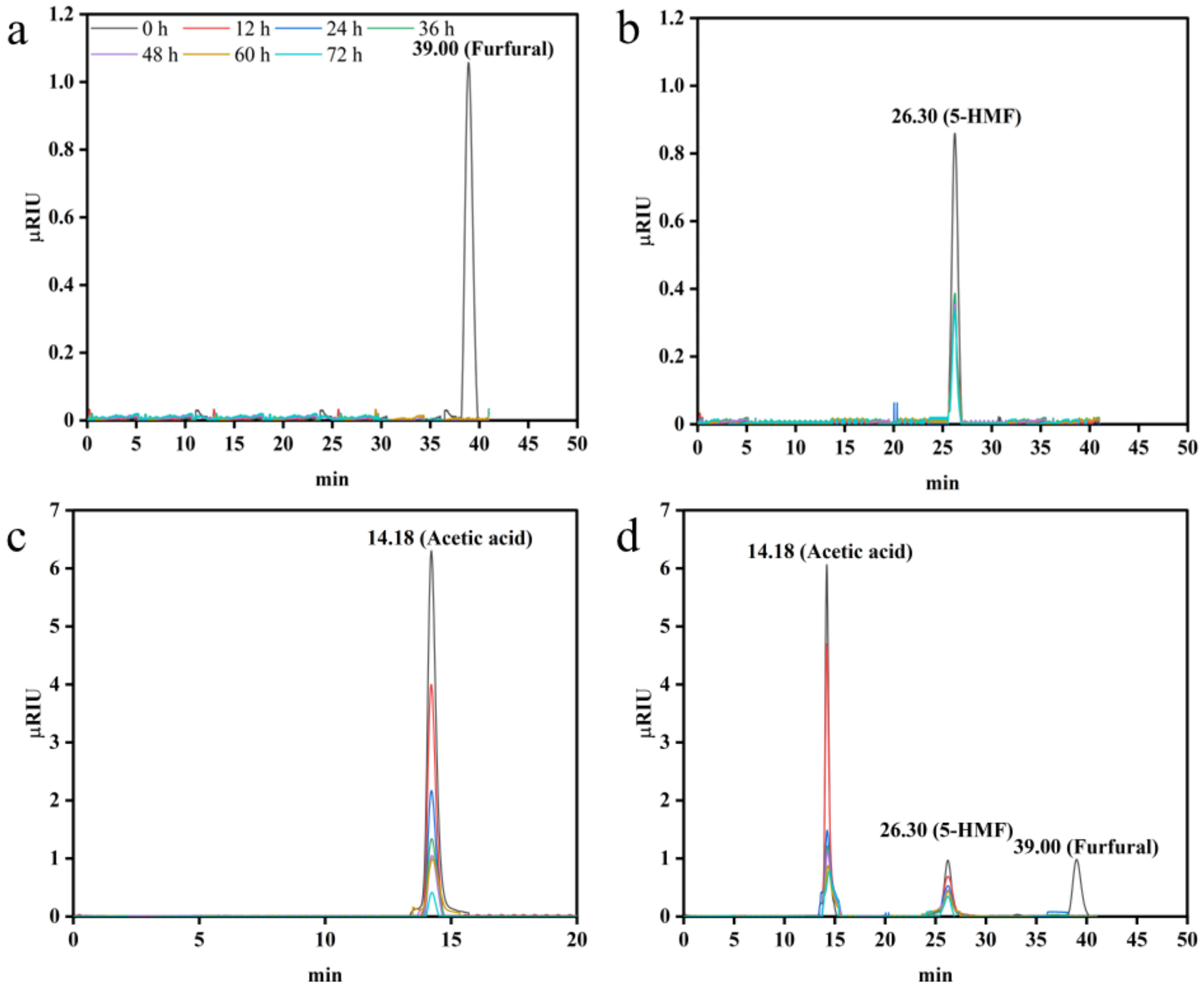

Figure S3: HPLC quantitative characterization of furfural, 5-HMF and acetic acid in fermentation processes with single and mixed conditions (low concentration). (a: Furfural at each time point from 0 to 72 h; b: 5-HMF at each time point from 0 to 72 h; c: Acetic acid at each time point from 0 to 72 h; d: Mixed inhibition of furfural, 5-HMF and acetic acid from 0 to 72 h).

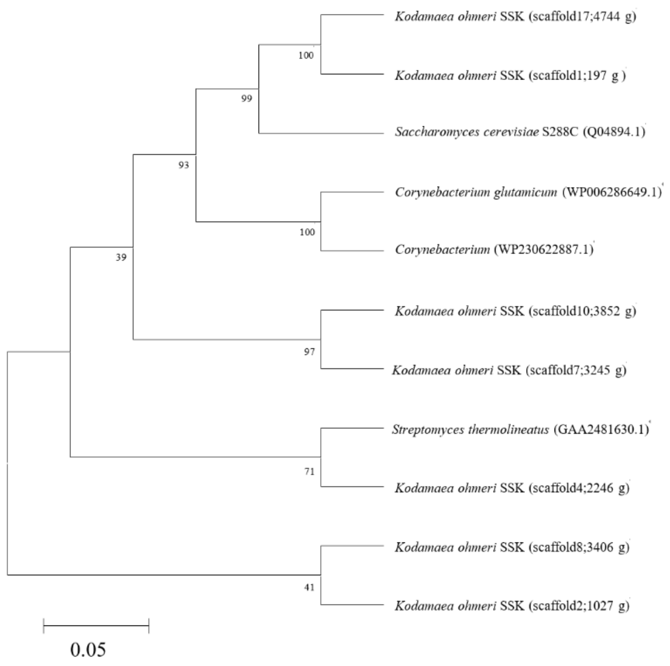

Figure S4: Phylogenetic tree of *K. ohmeri* SSK and multiple strains constructed based on amino acid sequences of alcohol dehydrogenase (ADH). This phylogenetic tree was constructed using Mega7.0 software. It includes ADH amino acid sequences from different species, such as the *S. cerevisiae* S288C strain, two sequences WP_006286649.1 and WP_230622887.1 from *Corynebacterium glutamicum*, the GAA2481630.1 sequence from *Streptomyces thermolineatus*, and sequences like scaffold17:4744_g and scaffold1:197_g in the genome of *K. ohmeri* SSK.

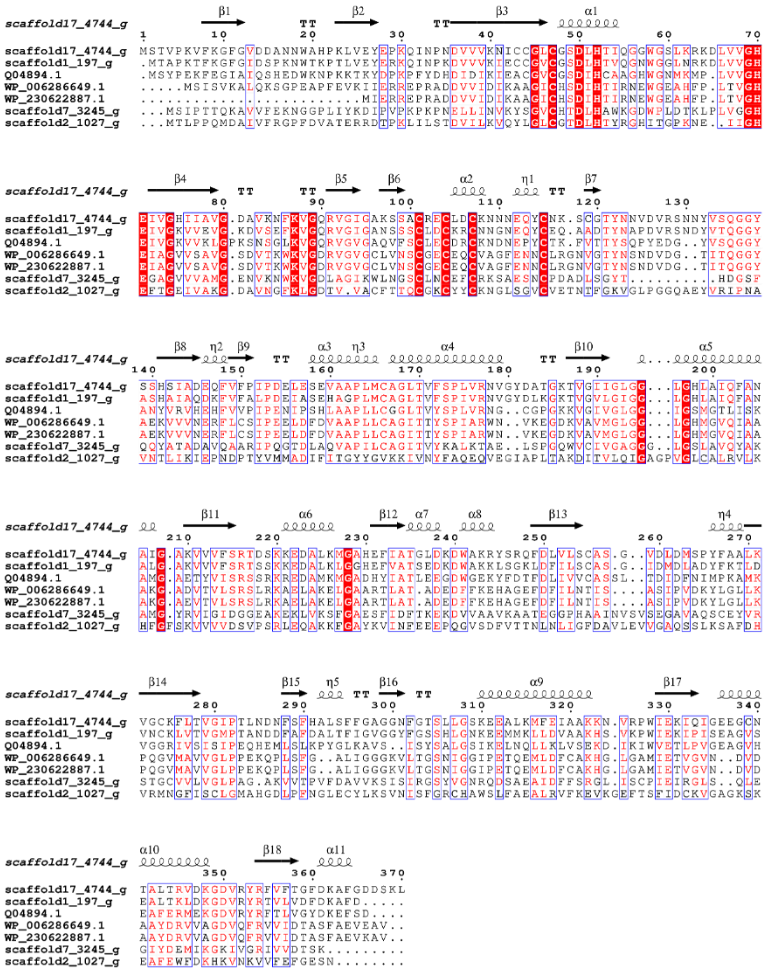

Figure S5: Multiple sequence alignment of amino acid sequences encoding alcohol dehydrogenase (ADH) from different species. The sequences are arranged vertically, and the amino acid positions are marked by numbers. Conserved amino acid residues are in red. Amino acids with similar function properties are in blue. Some sequences contain "-" gaps to better align sequences at different lengths.
